# The secreted MSP domain of *C. elegans* VAPB homolog VPR-1 patterns the adult striated muscle mitochondrial reticulum via SMN-1

**DOI:** 10.1242/dev.152025

**Published:** 2017-06-15

**Authors:** Jessica Schultz, Se-Jin Lee, Tim Cole, Hieu D. Hoang, Jack Vibbert, Pauline A. Cottee, Michael A. Miller, Sung Min Han

**Affiliations:** Department of Cell, Developmental and Integrative Biology, University of Alabama at Birmingham, Birmingham, AL 35294, USA

**Keywords:** MSP, Major sperm protein domain, SMN-1, VAPA, VAPB, Mitochondria, Striated muscle, ALS, Amyotrophic lateral sclerosis, SMA, Spinal muscular atrophy

## Abstract

The major sperm protein domain (MSPd) has an extracellular signaling function implicated in amyotrophic lateral sclerosis. Secreted MSPds derived from the *C. elegans* VAPB homolog VPR-1 promote mitochondrial localization to actin-rich I-bands in body wall muscle. Here we show that the nervous system and germ line are key MSPd secretion tissues. MSPd signals are transduced through the CLR-1 Lar-like tyrosine phosphatase receptor. We show that CLR-1 is expressed throughout the muscle plasma membrane, where it is accessible to MSPd within the pseudocoelomic fluid. MSPd signaling is sufficient to remodel the muscle mitochondrial reticulum during adulthood. An RNAi suppressor screen identified survival of motor neuron 1 (SMN-1) as a downstream effector. SMN-1 acts in muscle, where it colocalizes at myofilaments with ARX-2, a component of the Arp2/3 actin-nucleation complex. Genetic studies suggest that SMN-1 promotes Arp2/3 activity important for localizing mitochondria to I-bands. Our results support the model that VAPB homologs are circulating hormones that pattern the striated muscle mitochondrial reticulum. This function is crucial in adults and requires SMN-1 in muscle, likely independent of its role in pre-mRNA splicing.

## Abstract (precis)

**Highlighted Article:** Secreted MSPds promote the localization of mitochondria within the body wall muscle during development, with implications for ALS. See also the companion paper by Cottee et al.

## INTRODUCTION

VAMP/synaptobrevin-associated proteins (VAPs) comprise an evolutionarily conserved protein family with an N-terminal major sperm protein domain (MSPd), coiled-coil motif, and transmembrane region (Lev et al., 2008). They are synthesized as type II integral membrane proteins with the MSPd residing in the cytosol. The MSPd is named after nematode major sperm proteins, which function as sperm cytoskeletal elements and secreted signaling molecules (Bottino et al., 2002; Miller et al., 2001; Roberts and Stewart, 2012). In animals, VAPs have two diverse biochemical functions. VAPs act in a cell-autonomous fashion as scaffolding components at intracellular membrane contact sites (Lev et al., 2008; Stefan et al., 2011). In this capacity, there is evidence for roles in lipid transport, Ca^2+^ homeostasis, the unfolded protein response and other processes. VAPs also have a non-cell-autonomous signaling function (Han et al., 2012, 2013; Tsuda et al., 2008). In neurons and other cells, the MSPd is liberated from the transmembrane domain and unconventionally secreted into the extracellular environment. The MSPd signals through Eph and Lar-like receptors that modulate the actin cytoskeleton. An important MSPd target is striated muscle, where signaling regulates mitochondrial morphology and localization (Han et al., 2012, 2013).

Humans have two VAP paralogs called VAPA and VAPB, which have broad, largely overlapping expression patterns (Gkogkas et al., 2008; Larroquette et al., 2015; Lev et al., 2008). A P56S substitution in the VAPB MSPd is associated with amyotrophic lateral sclerosis (ALS) and spinal muscular atrophy (SMA) (Di et al., 2016; Nishimura et al., 2004). ALS clinical symptoms often emerge when the patient is in their fifties and are characterized by progressive muscle weakness, atrophy, and spasticity, resulting from degeneration of upper and lower motor neurons (Rowland, 1998). Most ALS cases occur sporadically without a clear family history. However, mutations in over 20 genes, including *VAPB*, are associated with familial ALS forms (Peters et al., 2015).

The MSPd P56S mutation also causes a late-onset form of SMA, which is characterized by lower motor neuron degeneration (Burghes and Beattie, 2009). Although SMA is more common in infants and children, rare adult-onset cases do occur (Nishimura et al., 2004; Tiziano et al., 2013). Reduced survival of motor neuron 1 (SMN-1) function causes ∼95% of all SMA cases (Burghes and Beattie, 2009; Lefebvre et al., 1995). SMN-1 is part of a protein complex that controls the assembly of small nuclear ribonucleoproteins (snRNPs) essential for pre-mRNA splicing (Fallini et al., 2012; Liu et al., 1997). It is not clear whether this function or an alternative function is crucial for SMA pathogenesis (Cauchi, 2010). For instance, SMN-1 localizes to myofilaments in *Drosophila* flight muscles, where it regulates actin dynamics (Rajendra et al., 2007).

Evidence is accumulating that MSPd signaling may be important in sporadic ALS cases. The VAP MSPd is found in human blood and cerebrospinal fluid (CSF), although its circulating function is not understood (Deidda et al., 2014; Tsuda et al., 2008). In an Italian cohort, a majority of sporadic ALS patients had undetectable VAPB MSPd levels in CSF (Deidda et al., 2014). The pathogenic P56S mutation prevents MSPd secretion in cultured cells and animal tissues (Han et al., 2012; Tsuda et al., 2008). EphA4, an ephrin receptor that also interacts with the VAPB MSPd (Lua et al., 2011; Tsuda et al., 2008), modifies pathogenesis in ALS patients and in a zebrafish model (Van Hoecke et al., 2012). Eph and Lar-related receptors are expressed in motor neurons and striated muscles. While both cell types are implicated in ALS, their respective roles are not well delineated (Dupuis et al., 2011; Turner et al., 2013; Zhou et al., 2010). Familial ALS patients carry the pathogenic mutation throughout their lives. Disease-causing mutant proteins tend to be expressed early and ubiquitously, potentially triggering secondary effects and compensatory mechanisms that mask the primary pathological event. Unfortunately, defining early pathogenic processes has proven challenging. A better understanding of MSPd function might provide insight into these processes.

*C. elegans* and *Drosophila* VAPs have an important signaling function that impacts striated muscle mitochondria (Han et al., 2012, 2013; Tsuda et al., 2008). MSPd signaling to *C. elegans* body wall muscle remodels the actin cytoskeleton, thereby docking mitochondria to myofilaments, altering fission/fusion balance and promoting energy metabolism (Han et al., 2012). MSPd antagonizes signaling via the CLR-1 Lar-related tyrosine phosphatase receptor. Excess CLR-1 activity promotes actin filament formation in the muscle belly, displacing mitochondria from I-bands. In aging worms, muscle cytoskeletal or mitochondrial abnormalities induce elevated Forkhead Box O (FoxO) transcription factor activity (Han et al., 2013). FoxO promotes muscle triacylglycerol (TAG) accumulation, alters ATP metabolism, and extends lifespan, despite reduced mitochondria electron transport chain activity. *Vapb* knockout mice also exhibit abnormal muscular FoxO metabolic gene regulation (Han et al., 2013). These data support the model that the MSPd promotes striated muscle energy metabolism.

Here we use *C. elegans* to further investigate the VAP-related 1 (VPR-1) signaling mechanism. Our results support the model that neurons and germ cells secrete the MSPd into the pseudocoelom, where it acts on CLR-1 receptors expressed throughout the muscle plasma membrane. Although *vpr-1* mutant muscle mitochondrial defects initiate early in larval development, MSPd–to–CLR-1 signaling is sufficient during the L4 stage and adulthood to localize mitochondria to I-bands. In a suppressor screen, we identified SMN-1 as a crucial MSPd downstream mediator in muscle, where it regulates mitochondrial morphology and localization. We propose that VAPB homologs have an evolutionarily conserved signaling function to pattern the mitochondrial reticulum in striated muscle. This signaling activity is essential during adulthood and requires SMN-1 in muscle.

## RESULTS

### Muscle mitochondrial defects in *vpr-1* mutants emerge in larval development

**Fig. 1.**
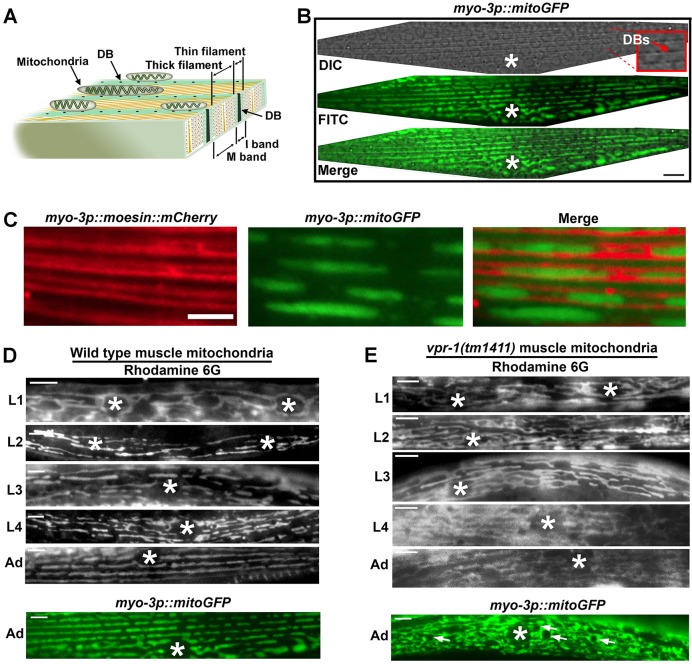
**Mitochondrial organization in *C. elegans* body wall muscle.** (A) Diagram of adult muscle myofilaments showing positions of mitochondria relative to I-bands. Mitochondrial tubules lie on top (or beneath, depending on orientation) of dense bodies (DBs). (B) Mitochondria in a single adult body wall muscle visualized with the *myo-3p::mitoGFP* transgene (Labrousse et al., 1999). Dense bodies are visible as dark dots along the muscle striations in the differential interference contrast (DIC) channel (arrow in higher magnification inset). FITC, fluorescein isothiocyanate. (C) Organization of mitochondria along thin filaments. The *myo-3p::moesin::mCherry* transgene labels muscle actin. Mitochondrial tubules extend along thin filaments, undergoing fission and fusion with adjacent tubules (Han et al., 2012). (D,E) Developmental timecourse of mitochondrial organization in wild-type (D) and *vpr-1(tm1411)* null mutant (E) muscles. Mitochondria are visualized by mitoGFP and Rhodamine 6G. Arrows indicate fat droplets; asterisk, nucleus; L1-L4, larval stages; Ad, adult stage. Scale bars: 5 µm.

In adult central body wall muscles, mitochondrial tubules lie in parallel arrays on top of (or beneath, depending on dorsal or ventral orientation) dense bodies along myofilament I-bands (Fig. 1A,B). Muscle mitochondria are visualized using a mitoGFP reporter expressed under the muscle-specific *myo-3* promoter (Fig. 1B), as well as dyes such as Rhodamine 6G and MitoTracker CMXRos (Han et al., 2012, 2013). Mitochondria localize along actin-rich thin filaments (Fig. 1C), where they undergo fission and fusion with adjacent tubules (Han et al., 2012). Myofilaments appear normal in *vpr-1* mutants, but ectopic Arp2/3-dependent actin network reorganization in the adult muscle belly displaces most mitochondria from I-bands (Han et al., 2012, 2013).

To investigate the origin of *vpr-1* mutant mitochondrial defects, we conducted a developmental timecourse starting at the L1 larval stage. Shortly after hatching, mitochondria in wild-type body wall muscle are predominantly peri-nuclear and extend thin tubules into the muscle cytoplasm or belly (Fig. 1D). As larvae develop, muscle mitochondria become progressively associated with I-bands, forming parallel arrays of mitochondrial tubules. By the early adult stage, most tubules are closely associated with I-bands (Fig. 1D) (Han et al., 2012). Thus, mitochondria in muscle develop their stereotypical positioning during larval development.

At hatching, muscle mitochondria in *vpr-1(tm1411)* mutants look similar to those in the wild type. The *tm1411* allele is a molecular null mutation that deletes the translational start site and entire MSPd (Han et al., 2012, 2013; Tsuda et al., 2008). *vpr-1* mutant mitochondria are predominantly peri-nuclear with tubules branching into the cytoplasm (Fig. 1E). However, mitochondria in *vpr-1* mutant muscles fail to target I-bands during larval development and adulthood. They largely remain in the muscle belly, forming branched networks as the muscle grows in size (Fig. 1E). These mitochondrial networks increase in complexity as the worm ages. Rhodamine 6G and MitoTracker CMXRos dyes stain *vpr-1(tm1411)* mutant muscle mitochondria well during early larval development. Staining is less efficient at the L4 and adult stages, perhaps reflecting changes in transmembrane potential or tubule architecture (Han et al., 2012). During adulthood, mitochondrial tubules are hyperfused and fat droplets accumulate in the belly (Han et al., 2012, 2013). Consistent with fission/fusion imbalance, transgenic lines expressing the fission mediator DRP-1::mCherry and fusion mediator FZO-1::mCherry show abnormal localization in adult *vpr-1* mutant muscle (Fig. S1). We conclude that the *vpr-1* mutant muscle mitochondrial defects initiate in larval development, resulting in abnormal mitochondrial fission/fusion dynamics in adults.

In neurons, synapses have a high energy demand that depends on closely associated mitochondria (Hollenbeck and Saxton, 2005). To determine if *vpr-1* loss affects neuronal mitochondrial localization, we generated wild-type and *vpr-1(tm1411)* transgenic lines expressing mitoGFP and the synaptic vesicle marker mCherry::RAB-3 (Ding et al., 2007; Nonet et al., 1997) in motor neurons. There was no statistical difference between the two lines in the percentage of mitochondria associated with synapses (Fig. S2). We observed a possible increase in mCherry::RAB-3 puncta size in *vpr-1(tm1441)* mutants, suggesting that synaptic size or vesicle density is increased. Therefore, *vpr-1* loss causes mitochondrial localization defects in larval and adult muscles, but not in motor neurons. Whether *vpr-1* loss causes functional or subtle trafficking defects in neuronal mitochondria is not clear with the present data.

### The nervous system and germ line are major origins of VPR-1 signaling activity

**Fig. 2.**
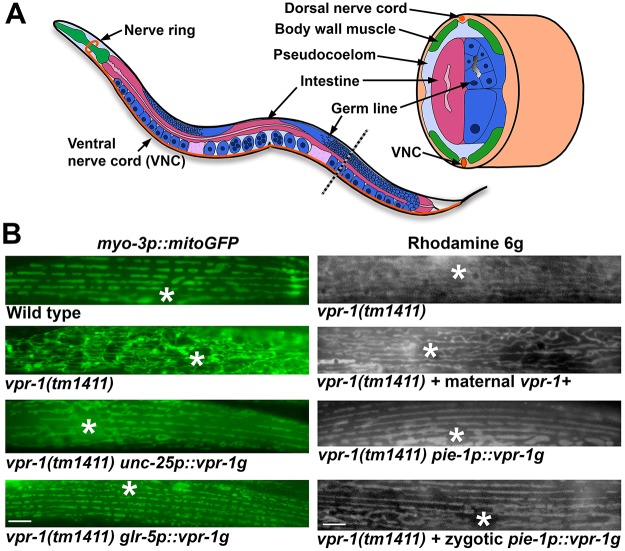
**Non-cell-autonomous VPR-1 action on body wall muscle mitochondria.** (A) Diagram of an adult hermaphrodite showing the germ line, nervous system, muscles and pseudocoelom (adapted from Altun and Hall, 2017). The dotted line indicates the level of the transverse section. (B) Mitochondria in adult body wall muscle visualized using mitoGFP or Rhodamine 6G. The *unc-25p::vpr-1g* transgene expresses *vpr-1* in GABA motor neurons along the nerve cord, whereas the *glr-5p::vpr-1* transgene expresses *vpr-1* in head interneurons mainly in the nerve ring. The pan-neuronal *unc-119* promoter, sensory neuron *osm-6* promoter, and cholinergic motor neuron *unc-17* promoter produced similar results (not shown). Maternal *vpr-1* is provided in oocytes from a *vpr-1(tm1441)/hT2* heterozygous hermaphrodite. The *pie-1p::vpr-1g* integrated single-copy (MosSCI) transgene expresses *vpr-1* in the germ line. Two independent *vpr-1(tm1411); Si1[pie-1p::vpr-1+unc-119(+)]* lines showed complete rescue of gonad and muscle phenotypes and were maintained as homozygous mutant lines. *vpr-1(tm1441)* hermaphrodites expressing *vpr-1* in the zygotic germ line only were generated by crossing *vpr-1(tm1411); Si1[pie-1p::vpr-1+unc-119(+)]* males to maternally rescued F1 *vpr-1(tm1441)* hermaphrodites. Asterisk, nucleus. Scale bars: 5 µm.

Previous experiments showed that driving *vpr-1* cDNA expression pan-neuronally using the *unc-119* promoter rescued ∼30-40% of the muscle mitochondrial defects in *vpr-1(tm1411)* mutants (Han et al., 2012). We found that *vpr-1* genomic sequence, including the 3′ UTR, is more efficient than the cDNA with the *unc-54* 3′ UTR in rescuing the *vpr-1* mutant gonadogenesis defect (Cottee et al., 2017). Transgenes containing *vpr-1* genomic DNA driven by pan-neuronal (*unc-119p*), GABA motor neuron (*unc-25p*), cholinergic motor neuron (*unc-17p*), head interneuron (*glr-5p*) or sensory neuron (*osm-6p*) specific promoters rescue the *vpr-1* mutant muscle mitochondrial defects in about half the muscles, with variation apparent among animals (Fig. 2; data not shown). By contrast, the endogenous *vpr-1* promoter and genomic locus provide complete rescue (Han et al., 2013). These results indicate that *vpr-1* expression in diverse neuron classes is sufficient to promote muscle mitochondrial localization, but additional sources of VPR-1 might be involved.

Genetic mosaic analysis using the *vpr-1* genomic locus indicates that *vpr-1* expression is essential in the nervous system and germ line but not in muscle to promote muscle metabolism (Han et al., 2013). The germ line is a source of maternal mRNAs provided to the embryo, as well as zygotic gene expression in developing and adult gonads. The germ line and muscle can exchange signaling molecules and other factors through the pseudocoelom (Fig. 2A). Maternal *vpr-1* mRNA is sufficient to weakly rescue the *vpr-1(tm1411)* mitochondrial defects in adult muscle (Fig. 2B). To investigate zygotic germline expression, we generated single-copy integrated transgenic lines that express *vpr-1* under the *pie-1* germline promoter (Seydoux and Dunn, 1997). Two independent integrated transgenes were crossed into the *vpr-1(tm1411)* background. Both transgenes completely rescued the muscle mitochondria (Fig. 2B) and gonad development defects (Cottee et al., 2017). We used two strategies to evaluate zygotic germline *vpr-1* expression (see Materials and Methods). These *vpr-1* mutant hermaphrodites lack maternal *vpr-1* mRNA and contain a single copy of the *pie-1p::vpr-1* transgene provided in the paternal genome. In both experiments, the *vpr-1* mutant muscle mitochondrial defects were rescued in about half the muscle, with variability among animals (Fig. 2B). Therefore, zygotic germline *vpr-1* expression is sufficient to promote body wall muscle mitochondrial localization. In summary, the results of these genetic mosaic and transgenic expression studies are consistent with the nervous system and germ line acting together as major sources of VPR-1 MSPd signaling activity to body wall muscle.

### Endogenous CLR-1 receptor is expressed throughout the sarcolemma in larval and adult worms

**Fig. 3.**
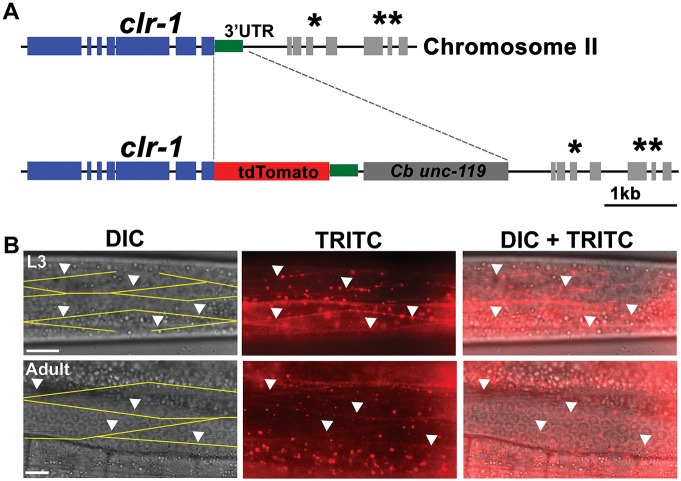
**Endogenous CLR-1 receptor expression in larval and adult muscles.** (A) Cas9 engineering showing the tdTomato insertion site within the *clr-1* genomic locus. The *C. briggsae unc-119* gene was used for screening. Asterisks indicate downstream genes (*, *mrps-16*; **, *F56D1.2*)*.* (B) DIC, fluorescence [tetramethylrhodamine isothiocyanate (TRITC)] and merged images focusing on body wall muscles. In the DIC image, the characteristic rhomboid shapes of body wall muscles are hand drawn in yellow (Francis and Waterston, 1985). Arrowheads point down to locations of *clr-1::tdTomato* expression along the muscle sarcolemma. Scale bars: 10 µm.

The VPR-1 MSPd might signal to muscle from the pseudocoelom or via the neuromuscular junction during larval development, adulthood, or both. MSPd signals are transduced in muscle through the CLR-1 receptor (Han et al., 2012, 2013). The CLR-1 expression pattern should provide clues as to where and when MSPd signal transduction initiates. To determine endogenous CLR-1 expression, we used Cas9 to fuse tdTomato to the *clr-1* genomic locus, creating a C-terminal fusion protein (Fig. 3A). Reduced *clr-1* function in the hypodermis causes fluid to accumulate throughout the pseudocoelom, arresting development and causing gonad degeneration (Huang and Stern, 2004; Kokel et al., 1998). tdTomato insertion did not disrupt CLR-1 function, as shown by growth to adulthood, fertility, and the absence of fluid accumulation. We observe endogenous CLR-1::tdTomato expression in muscle and a variety of other cell types, including somatic gonad, hypodermis and neurons (Fig. 3B and Fig. S3). In larval and adult body wall muscle, CLR-1 is expressed throughout the plasma membrane, called the sarcolemma, and in puncta within the muscle cytoplasm (Fig. 3B). We did not detect enrichment or absence at post-synaptic plasma membrane sites near the nerve cord, showing that CLR-1 is uniformly expressed. Cell surface CLR-1 expression did not significantly change in the *vpr-1(tm1411)* background (Fig. S4). Therefore, the CLR-1 extracellular domain is accessible to MSPd signals secreted from adjacent motor neurons or from more distant neurons and germ cells via the pseudocoelom.

### MSPd signaling via CLR-1 is sufficient during adulthood

**Fig. 4.**
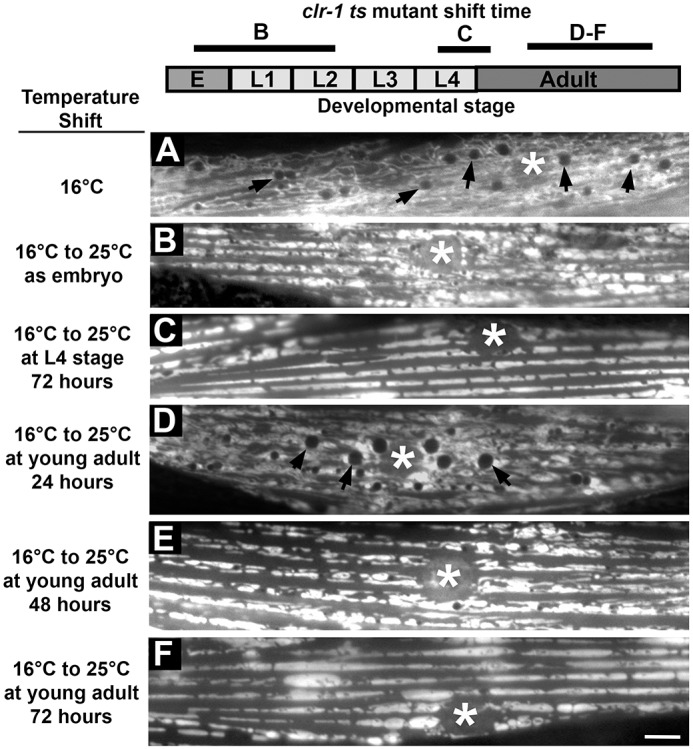
**Temporally controlled MSPd activity in *vpr-1* null mutants.** The *clr-1(e1745ts)* temperature-sensitive mutation was used to inactivate CLR-1 (Kokel et al., 1998), thereby simulating MSPd signaling. The *rol-6p::clr-1* transgene drives *clr-1* expression specifically in the hypodermis to alleviate fluid accumulation (Huang and Stern, 2004). *myo-3p::mitoGFP* was used to visualize muscle mitochondria. The diagram summarizes the restrictive temperature shift initiation period according to developmental stage. E, embryonic stage. (A) *vpr-1(tm1411);clr-1(e1745ts)* adult worm grown at the permissive temperature. Mitochondrial tubules are found throughout the muscle belly, along with fat droplets (arrows) (Han et al., 2013). In some animals, the mitochondria appear fragmented. (B) *vpr-1(tm1411);clr-1(e1745ts)* adult worm shifted to restrictive temperature as embryos. The mitochondria largely align at I-bands, but are often fragmented and poorly organized. Few fat droplets are seen. Similar results are observed when shifting at L1 and L2 stages. (C) *vpr-1(tm1411);clr-1(e1745ts)* adult worm shifted to restrictive temperature for 3 days starting as an L4. (D-F) Timecourse showing *vpr-1(tm1411);clr-1(e1745ts)* adult worm shifted to the restrictive temperature for (D) 24 h, (E) 48 h and (F) 72 h. Asterisk, nucleus. Scale bar: 10 µm.

The MSPd interacts with the CLR-1 extracellular domain to antagonize CLR-1 receptor tyrosine phosphatase signaling (Han et al., 2012). Accordingly, CLR-1 loss suppresses the *vpr-1(tm1411)* mitochondrial defects (Han et al., 2012). To investigate the temporal requirements of MSPd signaling, we took advantage of the *clr-1(e1745ts)* temperature-sensitive allele (Kokel et al., 1998). Shifting *clr-1(e1745ts)* worms from the permissive temperature (16°C) to the restrictive temperature (25°C) reduces CLR-1 function, resulting in fluid accumulation. To avoid confounding issues due to fluid, we generated *vpr-1(tm1411); clr-1(e1745ts)* transgenic animals that express *clr-1* specifically in the hypodermis using the *rol-6* promoter. The *rol-6p::clr-1* transgene largely rescued the *clr-1(e1745ts)* fluid accumulation defect, allowing for development (Fig. S5). At the permissive temperature, muscle mitochondria in transgenic double mutants exhibited either the highly branched networks seen in *vpr-1* null muscle or more fragmented networks with no apparent organization (Fig. 4A). We shifted the double mutants to the restrictive temperature at various times during development, thereby simulating MSPd signaling through CLR-1 inactivation. When the mutants were shifted at embryonic or early larval stages (L1-L2) and grown to adulthood, mitochondria aligned at myofilaments, but with abnormal morphology (Fig. 4B). However, shifting them at L4 and early adulthood rescued the muscle mitochondrial defects scored 3 days later (Fig. 4C). These data suggest that MSPd signaling via CLR-1 is sufficient in late larval and adult stages, when a major source of MSPd signals, the germ line, is expanding and differentiating. Temporally inducing *vpr-1* expression in head neurons using the binary Q system (Wei et al., 2012) also promoted muscle mitochondrial alignment (Cottee et al., 2017).

In 1-day adult *vpr-1* mutants, the muscle mitochondrial reticulum is strongly disrupted due to excess CLR-1 activity (Fig. 1E) (Han et al., 2012). To test whether this reticulum can be remodeled, we shifted 1-day adult worms to the restrictive temperature and observed their mitochondria 24, 48 and 72 h later. After 24 h, the mitochondria are still largely disorganized and surround fat droplets in the muscle belly (Fig. 4D). Mitochondria disorganization and increased fat droplets are both due to abnormal Arp2/3 activity (Han et al., 2012, 2013). Shifting for 24 h is sufficient to induce fluid accumulation in nontransgenic mutants, indicating that CLR-1 function is compromised. At 48 h, most mitochondrial tubules are aligned at myofilaments, although morphology is still abnormal (Fig. 4E). By 72 h, mitochondria are positioned correctly at I-bands with morphology similar to those in wild-type muscle (Fig. 4F). Very few fat droplets are observed in the cytoplasm. These data suggest that MSPd signals continuously instruct muscle mitochondria to remodel via an active process throughout adulthood.

### A *vpr-1* mutant mitochondrial suppressor screen identifies *smn-1*

**Fig. 5.**
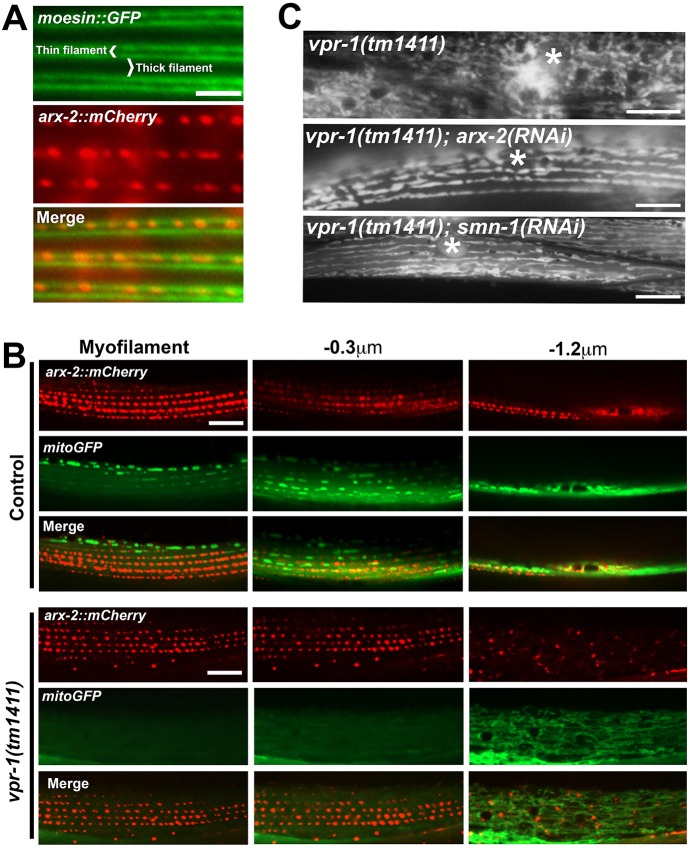
**Arp2/3 complex localization and function in wild-type and *vpr-1* mutant muscles.** (A) ARX-2 localization at the muscle myofilament, visualized using a functional *myo-3p::arx-2::mCherry* transgene and *myo-3p::moesin::GFP* transgene that labels actin. ARX-2::mCherry puncta lie between thin filaments, possibly at dense bodies. (B) ARX-2::mCherry and mitoGFP localization in adult wild-type and *vpr-1* mutant muscle. Columns show different depths of view, starting at the myofilament. The muscle belly lies 1-3 μm beneath myofilaments. (C) Representative results from the suppressor screen showing *arx-2* RNAi and *smn-1* RNAi. Asterisk, nucleus. Scale bars: 5 µm in A; 10 µm in B,C.

We next sought to understand how MSPd signals are transduced in muscle. To identify downstream mediators, we developed a *vpr-1(tm1411)* RNAi suppressor screen based on prior work using *arx-2* and *clr-1*. *arx-2* (also known as *arp-2*) encodes a component of the Arp2/3 complex (Roh-Johnson and Goldstein, 2009). Arp2/3 loss in *vpr-1* mutants suppresses the muscle mitochondrial defects (Han et al., 2012). Transgenic lines that express functional ARX-2::mCherry (see below) in wild-type muscle show punctate localization between thin filaments, slightly above mitochondrial tubules (Fig. 5A,B). Little ARX-2::mCherry is found in the muscle belly (Fig. 5B). In *vpr-1* mutants, ARX-2::mCherry is observed at myofilaments and throughout the belly (Fig. 5B), where actin filaments and most mitochondria exist. These data support the model that excess CLR-1 signaling promotes Arp2/3 activity and/or localization in the muscle cytoplasm, preventing mitochondria from targeting I-bands. Disrupting this pathway suppresses the *vpr-1(tm1411)* muscle mitochondrial defects, which is likely to be because a redundant mechanism localizes mitochondria to I-bands.

Our suppressor screen should identify those gene products specifically required for abnormal mitochondrial localization caused by MSPd deficiency. As a pilot investigation, we tested 31 RNAi clones corresponding to *C. elegans* homologs of genes implicated in ALS and SMA (Table S1). Three potential suppressors were identified: the nuclear export receptor *xpo-1*, the *Gemin3* homolog *mel-48*, and the survival of motor neuron 1 gene *smn-1*. In this paper, we focus on *smn-1*. Similar to inactivation of *clr-1* or *arx-2* (Fig. 4D and Fig. 5C), *smn-1* RNAi largely suppresses the muscle mitochondrial defects in *vpr-1* mutant animals (Fig. 5C). *smn-1* RNAi initiated in the parental generation often causes larval arrest in *vpr-1* mutant progeny and muscle mitochondria to localize to I-bands with more globular morphology. *smn-1* RNAi initiated in L2-L3 larva is sufficient to partially suppress the *vpr-1* mutant mitochondrial defect, suggesting that *smn-1* activity is essential during late larval development and adulthood. *smn-1* RNAi also partially suppresses *vpr-1* null mutant muscle fat droplet accumulation, as measured using fluorescent BODIPY fatty acid analogs (Fig. S6). We did not observe mitochondrial or fat droplet suppression following RNAi of *C. elegans* homologs of *Gemin2*, *Gemin6*, *Gemin7* or other genes crucial for pre-mRNA splicing (Table S1 and Fig. S7). Hence, *smn-1* function may be independent of its role in spliceosome assembly.

**Fig. 6.**
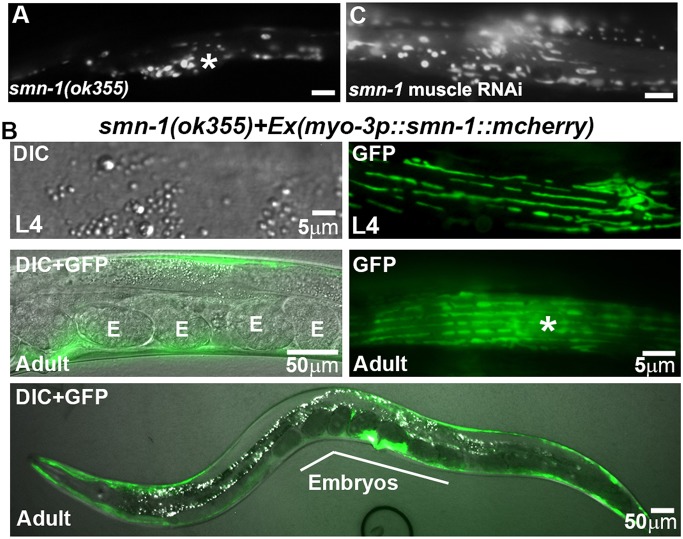
**SMN-1 functions in body wall muscle.** (A) Mitochondria visualized with mitoGFP in *smn-1(ok355)* mutant muscle. *smn-1(ok355)* hermaphrodites arrest development around the L3 stage when maternal *smn-1* runs out (Briese et al., 2009). (B) Muscle mitochondria in *smn-1(ok355)* mutants expressing *smn-1::mCherry* specifically in muscle. The top two panels are from the same L4 stage hermaphrodite; the bottom three panels are from different adult hermaphrodites. Some transgenic mutants are able to progress to L4 and young adult stages and contain embryos (E), which are not seen in nontransgenic *smn-1(ok355)* mutants. (C) Mitochondria in adult muscle following muscle-specific *smn-1* RNAi using *sid-1* mutants. Asterisk, nucleus. Scale bars: 5 µm in A,C; as indicated in B.

*smn-1* is broadly expressed and *smn-1(ok355)* null mutants arrest development around the L3 stage, after maternal *smn-1* mRNA is depleted (Briese et al., 2009). During this stage, mitochondria in *smn-1* knockout body wall muscle are largely globular in morphology and abnormally positioned throughout the belly (Fig. 6A). To test whether *smn-1* functions in muscle, we generated *smn-1(ok355)* transgenic worms that express *smn-1::mCherry* specifically in muscle using the *myo-3* promoter. The *myo-3p::smn-1::mCherry* transgene rescued the muscle mitochondrial defects of *smn-1(ok355)* worms (Fig. 6B). In addition, muscle *smn-1* expression partially suppresses the larval arrest and sterility phenotypes in a subset (<15%) of *smn-1(ok355)* mutants (Fig. 6B). We conclude that the *smn-1::mCherry* transgene is functional and *smn-1* expression in muscle is sufficient to regulate mitochondria.

To test whether *smn-1* is necessary in muscle, we specifically depleted *smn-1* in body wall muscle of wild-type animals using an RNAi mosaic strategy (Durieux et al., 2011; Esposito et al., 2007). *sid-1* (systemic RNA interference deficiency-1) mutants are defective for siRNA transport between cells, thereby preventing systemic RNAi effects. However, producing siRNA within tissues still induces cell-autonomous RNAi (Winston et al., 2002). We expressed *smn-1* sense and antisense RNAs in body wall muscle of *sid-1(pk3321)* mutants. Muscle *smn-1* RNAi causes mitochondrial morphological defects similar to those seen in *smn-1* mutants or systemic RNAi hermaphrodites (Fig. 6A,C). *smn-1* reduction of function suppresses the *vpr-1* null muscle mitochondrial defects, whereas *smn-1* loss throughout development causes more globular mitochondrial morphology. In summary, a *vpr-1* mitochondrial suppressor screen identified *smn-1*, which is necessary and sufficient in muscle to control mitochondrial morphology.

### SMN-1 and ARX-2 colocalize at muscle myofilaments

**Fig. 7.**
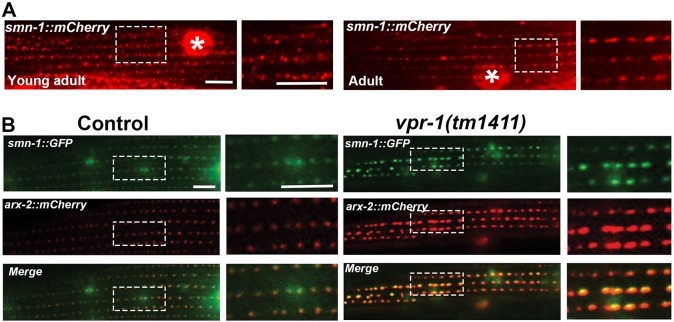
**SMN-1 and ARX-2 localization in body wall muscle myofilaments.** (A) The rescuing *myo-3p::smn-1::mCherry* transgene shows SMN-1 expression in the nucleus (asterisk) and in puncta at myofilaments. Expression is sometimes observed in peri-nuclear puncta in the muscle belly. (B) Confocal images showing SMN-1::GFP and ARX-2::mCherry localization at myofilaments of adult control and *vpr-1* mutant muscles. Boxed areas are magnified on the right. Puncta are also observed in the muscle belly of *vpr-1* mutants (see Fig. 5B). Scale bars: 5 µm.

SMN-1 expressed from the rescuing *smn-1::mCherry* transgene is seen in the nucleus and cytoplasm (Fig. 7A). In the cytoplasm, most SMN-1::mCherry is found at myofilaments in regularly spaced puncta. These puncta appear more ordered in adult muscle as compared with younger muscle (Fig. 7A). The SMN-1 expression pattern at muscle myofilaments resembles that of ARX-2::mCherry (Fig. 5A). To test whether ARX-2 and SMN-1 colocalize, we generated transgenic animals expressing both *smn-1::GFP* and *arx-2::mCherry* in muscle. SMN-1::GFP colocalizes with ARX-2::mCherry at the thin filaments (Fig. 7B). However, only SMN-1 is observed in the nucleus. We occasionally observed SMN-1::GFP and ARX-2::mCherry in peri-nuclear puncta (data not shown). SMN-1::GFP and ARX-2::mCherry also colocalize in the *vpr-1(tm1411)* background, indicating that MSPd signaling does not influence their association (Fig. 7B). We conclude that SMN-1 and ARX-2 colocalize at muscle myofilaments adjacent to elongated mitochondrial tubules.

### SMN-1 may promote Arp2/3 activity in muscle

**Fig. 8.**
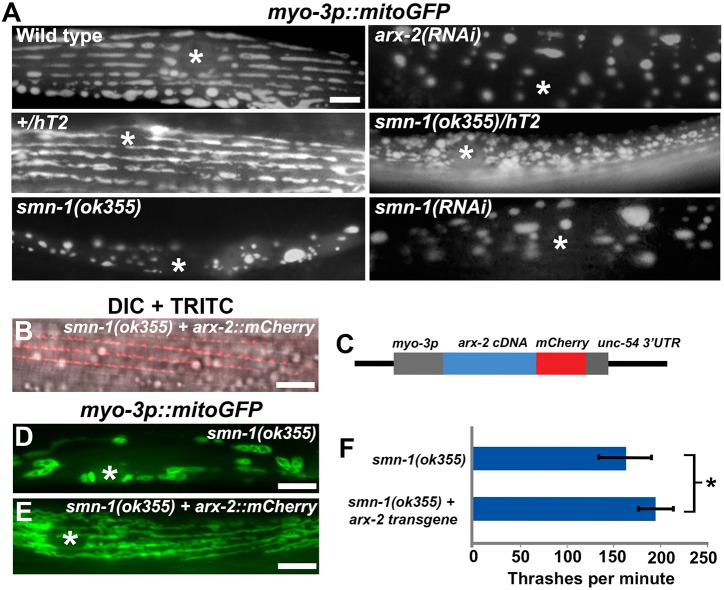
**SMN-1 promotes Arp2/3 activity in muscle.** (A) Muscle mitochondria in control, mutant, and RNAi hermaphrodites. *smn-1* reduction of function closely resembles *arx-2* reduction of function (Han et al., 2012). The hT2 balancer chromosome has a wild-type copy of *smn-1*. (B) Merged DIC and fluorescence (TRITC) image showing ARX-2::mCherry localization at muscle myofilament of an *smn-1(ok355)* mutant. (C) The *arx-2::mCherry* transgene. (D,E) Muscle mitochondria in nontransgenic (D) and transgenic (E) *smn-1(ok355)* mutants. Asterisk (A,D,E), nucleus. (F) Body bends per minute of L3 stage worms of the indicated genotypes in liquid medium (*N*=15). Bars indicate s.d. The wild-type value is 242±21 thrashes per minute. *P<0.01, Student's *t*-test. Scale bars: 5 µm.

The above data and dependence of mitochondrial localization on actin remodeling raise the possibility that SMN-1 promotes Arp2/3 activity. In this case, *smn-1* depletion should cause a similar muscle mitochondrial phenotype as *arx-2* depletion. Although *arx-2* null worms are embryonic lethal, restricting *arx-2* inactivation to wild-type larval and adult stages via RNAi feeding causes muscle mitochondria to adopt globular morphologies (Fig. 8A) (Han et al., 2012). Muscle mitochondria in *smn-1(ok355)* and *smn-1* RNAi hermaphrodites exhibit similar globular morphologies (Fig. 6 and Fig. 8A). Moreover, *smn-1(ok355)* heterozygotes also contain abnormally shaped mitochondria (Fig. 8A), suggesting that the *smn-1* expression level is important. Thus, reduced *smn-1* or *arx-2* function causes similar muscle mitochondrial phenotypes.

Current data suggest that *arx-2* and *smn-1* act in the same pathway. To further investigate their relationship, we expressed the *myo-3p::arx-2cDNA::mCherry* transgene in *smn-1(ok355)* null mutants (Fig. 8B-F). *arx-2* expression may escape endogenous RNA regulatory interactions because the transgene lacks *arx-2* introns and 3′ UTR (Fig. 8C). We found that ARX-2::mCherry localizes to myofilament puncta in *smn-1(ok355)* muscle (Fig. 8B), similar to the localization in wild-type muscle (Fig. 5). Importantly, ARX-2::mCherry overexpression partially suppresses the *smn-1* mutant mitochondrial defect, as mitochondria adopt elongated morphologies often along I-bands (Fig. 8D,E). ARX-2::mCherry overexpression also mildly, but significantly, increased *smn-1* mutant mobility in the thrashing assay (Briese et al., 2009), which measures the ability to contract body wall muscles in liquid medium (Fig. 8F). Taken together, the results support the model that SMN-1 and Arp2/3 promote actin remodeling events needed to localize mitochondria in muscle.

## DISCUSSION

The results presented here, together with those in prior studies, suggest that VAPB proteins have a noncanonical function as an endocrine factor to pattern the striated muscle mitochondrial reticulum. The primary mechanism hinges on a secreted VAP proteolytic fragment, the MSPd (Tsuda et al., 2008). In *C. elegans*, the secreted MSPd antagonizes the CLR-1 Lar-like phosphatase receptor, triggering changes in muscle mitochondrial localization, fission/fusion and function (Han et al., 2012). Disrupting MSPd signaling causes energy deficit in adult muscle, along with compensatory metabolic changes during aging (Han et al., 2013). Here we show that muscle mitochondria target myofilament I-bands during late larval development and adulthood. This mitochondrial patterning mechanism fails in *vpr-1* null mutants. Our results support the model that neurons and germ cells secrete the MSPd into the pseudocoelomic fluid, where it interacts with CLR-1 throughout the muscle plasma membrane. Downstream in the muscle cytoplasm, actin cytoskeletal reorganization events requiring the Arp2/3 complex and SMN-1 shift mitochondria from the cytoplasm to I-bands, where they form parallel arrays that fuse and divide. Continuous MSPd signaling during adulthood is required to maintain mitochondrial localization. Below, we further discuss the model, focusing on its developmental origin and implications for motor neuron diseases.

Genetic mosaic analysis demonstrates that *vpr-1* loss in the nervous system or germ line, but not in muscle, causes the muscle metabolic abnormalities (Han et al., 2013). Expressing *vpr-1* specifically in germ cells or neuron subsets is sufficient to rescue the *vpr-1* mutant muscle and gonad phenotypes, although not in all animals. Expression from multiple cell types provides complete rescue. The data point to neurons and germ cells as primary MSPd secretion sites, which act collectively. Secreted MSPds are likely to enter the pseudocoelom, a primitive circulatory system that bathes the body wall muscles and other internal organs (Hall et al., 1999). We show that endogenous CLR-1 receptor is expressed throughout the muscle plasma membrane in larval and adult worms, where it is accessible to the pseudocoelomic fluid. These results are consistent with the MSPd having endocrine and paracrine signaling activity to muscle.

A temperature-sensitive *clr-1* mutation (Kokel et al., 1998) was used to temporally induce MSPd signaling in *vpr-1* null animals. MSPd signaling at the L4 stage or during adulthood is sufficient to localize mitochondrial tubules to muscle I-bands. Earlier inductions promote mitochondrial localization, but morphology is abnormal. It is possible that MSPd levels rise throughout larval development as germ cells increase in number, further antagonizing CLR-1 signaling. In wild-type animals, reducing muscle *clr-1* function throughout larval development causes globular instead of tubular mitochondrial morphology (Han et al., 2012). A balance in CLR-1 signaling might be important early in development, whereas CLR-1 is largely inactive during adulthood. Muscle mitochondrial networks are already disorganized in young adult *vpr-1* mutants, yet CLR-1 inactivation at this time induces remodeling events that target mitochondria to I-bands within 48 to 72 h. Therefore, MSPd activity on muscle appears to be instructive.

In muscle, CLR-1 activity triggers actin remodeling in the muscle belly that is dependent on the Arp2/3 complex (Han et al., 2012). These actin networks prevent mitochondria from associating with actin-rich I-bands, where ATP and Ca^2+^ levels fluctuate (Moerman and Williams, 2006). The MSPd attenuates CLR-1 signaling, thereby restricting Arp2/3 activity to the I-band. Mitochondria then align along the I-band, alter their fission/fusion properties, and alter function (Han et al., 2013). Whether Arp2/3 restriction occurs by controlling localization or actin nucleation activity is not clear. We identified *smn-1* in an RNAi screen for *vpr-1* mutant mitochondrial suppressors. SMN-1 acts in muscle, where it colocalizes with the Arp2/3 complex. Genetic studies are consistent with SMN-1 promoting Arp2/3 activity. This function is sensitive to gene dosage, suggesting that the SMN-1 expression level is important. It might also require the Gemin3 homolog MEL-46, but appears independent of other gemins, SM proteins, and other proteins involved in snRNP assembly. Prior studies have implicated SMN-1 in mRNA transport and actin remodeling independent of its snRNP role, although the mechanism is not well understood (Burghes and Beattie, 2009; Fallini et al., 2012; Rajendra et al., 2007).

In humans, reduced VAPB MSPd function is associated with sporadic and familial ALS cases (Deidda et al., 2014; Kabashi et al., 2013; Larroquette et al., 2015; Mitne-Neto et al., 2011; Teuling et al., 2007; Tsuda et al., 2008). Patients with the VAPB P56S mutation present with ALS, atypical ALS forms, or late-onset SMA (Marques et al., 2006; Nishimura et al., 2004). Outside of these patients, the vast majority of late-onset (type IV) SMA cases are due to reduced *SMN1* function (Burghes and Beattie, 2009). Delayed onset is thought to result from extra copies of *SMN2*, which can weakly compensate for *SMN1* loss (Butchbach, 2016). *SMN1* and *SMN2* copy number have also been implicated in ALS (Blauw et al., 2012; Butchbach, 2016). Our results show that *C. elegans* VAPB and SMN-1 homologs act in a signaling pathway governing a striated muscle mitochondrial transition. Motor neurons expressing the MSPd/ephrin receptor VAB-1 might also participate in some way (Brisbin et al., 2009).

In mammalian fast-twitch muscle fibers, mitochondrial doublets encircle myofibers at I-bands, where they couple to the sarcotubular system (Boncompagni et al., 2009; Pham et al., 2012). Similar to *C. elegans*, mitochondria acquire this I-band positioning during postnatal development (Boncompagni et al., 2009). These data raise an intriguing model. Reduced VAPB or SMN-1 function might perturb a mitochondrial transition in the postnatal neuromuscular system. Motor neurons innervating fast-twitch fibers are the first to degenerate in ALS mouse models (Van Hoecke et al., 2012; Vinsant et al., 2013a,b). Either muscle mitochondrial dysfunction or compensatory mechanisms could predispose these motor neurons to degeneration during aging. An important implication is that motor neuron disease may initiate many years before clinical symptoms emerge. Therefore, a much larger window could exist for therapeutic interventions and biomarker development to track disease progression.

## MATERIALS AND METHODS

### *C. elegans* genetics and strains

*C. elegans* were maintained at 20°C unless otherwise indicated, and fed with NA22 *E. coli* bacteria (Brenner, 1974; Edmonds et al., 2010; Kubagawa et al., 2006). The following strains were used: N2 Bristol (wild type); CB3241 *clr-1(e1745ts)*; VC1478 *vpr-1(tm1411)/hT2 [bli-4(e937) let-?(q782) qIs48] (I;III)*; XM1101 *vpr-1(tm1411)/hT2 [bli-4(e937) let-?(q782) qIs48]; clr-1(e1745ts)*; XM1102 *clr-1(e2530)/mIn1 [dpy-10(e128) mIs14] (II)*; and LM99 *smn-1(ok355)/hT2 [bli-4(e937) let-?(q782) qIs48] (I;III)*.

Studies with the *clr-1(e1745ts)* temperature-sensitive (ts) allele were performed at permissive (16°C) and restrictive (25°C) temperatures. Strain construction involved PCR, sequencing (UAB Heflin Center for Genomics Sciences), and phenotypic analyses. *vpr-1(tm1441)* mutants are maternal effect sterile (Cottee et al., 2017). Phenotypes were evaluated in *vpr-1(tm1411)* homozygous F2 progeny from *vpr-1(tm1411)/hT2* heterozygotes (P0), unless otherwise indicated. *vpr-1(tm1411)* homozygous F1 progeny contain maternal *vpr-1* mRNA.

### Zygotic *vpr-1* germline expression

We used two strategies to evaluate zygotic germline *vpr-1* expression. In the first, we mated *vpr-1(tm1411); Si1[pie-1p::vpr-1+unc-119(+)]* males to F1 *vpr-1(tm1411)* hermaphrodites derived from *vpr-1(tm1411)/hT2* heterozygous hermaphrodites. All cross progeny were scored. These *vpr-1(tm1411)* null worms lack maternal *vpr-1* and contain a single copy of the male-derived *pie-1p::vpr-1* transgene for zygotic germline expression. In the second strategy, we mated *vpr-1(tm1411); Si1[pie-1p::vpr-1+unc-119(+)]* males to *vpr-1(tm1411); Ex[unc-119p::vpr-1+myo-3p::mitoGFP]* hermaphrodites. *vpr-1(tm1411)* cross progeny lacking the extrachromosomal array were scored. These progeny contained a single copy of the male-derived *pie-1p::vpr-1* transgene. Both experiments show rescue of the *vpr-1* mutant muscle mitochondrial defects in about half the muscle, with variability among animals.

### Molecular cloning

To create the Cas9 DNA template for tdTomato insertion into the *clr-1* genomic locus, Gibson assembly was used to construct a plasmid containing *clr-1* 2 kb left homology arm::tdTomato::*clr-1* 3′UTR::*unc-119*::2 kb right homology arm. The *unc-119* rescue fragment is from plasmid pCFJ66 (Addgene plasmid #24981) and includes *C. briggsae unc-119*. The single guide RNA (sgRNA) plasmid was derived from Addgene plasmid 46169. Cas9 targeting sequence was 5′-ACTATATCTCTAAGACATAT-3′. PCR was used to amplify the entire sgRNA backbone, except for 20 bp that belong to *unc-119*. DNA fusions were constructed using Gibson assembly. The *rol-6p::clr-1* construct was made with *clr-1* genomic DNA. 2 kb upstream of the *rol-6* start codon was amplified by PCR. *unc-119p::mitoGFP* was created using the *unc-119* promoter (Maduro and Pilgrim, 1995) and mitoGFP sequences (Labrousse et al., 1999). Pan-neuronal (*unc-119p*, 2000 bp), GABA motor neuron (*unc-25p*, 1893 bp) (Jin et al., 1999), cholinergic motor neuron (*unc-17p*, 2003 bp) (Lickteig et al., 2001), head interneuron (*glr-5p*, 2003 bp) (Brockie et al., 2001) and sensory neuron (*osm-6p*, 430 bp) (Collet et al., 1998) promoters were fused upstream of the *vpr-1* genomic locus, which included the 3′ UTR. PCR was used to amplify sequences from genomic DNA. The *pie-1* promoter sequence included 1095 bp upstream of the *pie-1* translational start site. The *vpr-1* DNA sequence included exons and introns, as well as 745 bp of 3′ UTR.

Gateway recombination (Invitrogen) was used to generate *myo-3p::moesin::mCherry*, *myo-3p::moesin::GFP*, *myo-3p::arx-2::mCherry*, *myo-3p::smn-1::mCherry*, *myo-3p::smn-1::GFP*, *myo-3p::drp-1::mCherry* and *myo-3p::fzo-1::mCherry.* To generate entry plasmids, *drp-1*, *fzo-1* and *arx-2* entry plasmids were prepared by PCR using a cDNA library as the template. *smn-1* cDNA was amplified by PCR from a plasmid containing the *smn-1* ORF (Open Biosystems). *Drosophila Moesin* (*dmoesin*) was amplified by PCR from pJWZ6 (Addgene plasmid #21744). All PCR products were cloned into pDONR221 entry vector and sequenced. The entry vectors were subsequently used for the LR recombination reaction. The *unc-54* 3′ UTR was used, except where indicated. Primers (5′-3′; F, forward; R, reverse) are: *drp-1* F1, GGGGACAAGTTTGTACAAAAAAGCAGGCTCCATGGAAAATCTCATTCCTGTCG; *drp-1* R1, GGGGACCACTTTGTACAAGAAAGCTGGGTACCAAACTTGTGTTTCTCTCAC; *fzo-1* F1, GGGGACAAGTTTGTACAAAAAAGCAGGCTCCATGTCTGGCACAGCAAGCTTA; *fzo-1* R1, GGGGACCACTTTGTACAAGAAAGCTGGGTATGGCGTTGGCGGAGAGTC; *arx-2* F, GGGGACAAGTTTGTACAAAAAAGCAGGCTCCATGGATTCGCAAGGGCGAAAG; *arx-2* R, GGGGACCACTTTGTACAAGAAAGCTGGGTAAGCTTTGATTCCAAGTTTGGC; *smn-1* F, GGGGACAAGTTTGTACAAAAAAGCAGGCTCCATGGCAAAAATCTGGTCGAAAAG; *smn-1* R, GGGGACCACTTTGTACAAGAAAGCTGGGTAATTTTGAACATTTTTCTGATCCGC; *arx-2* F2, GGGGACAGCTTTCTTGTACAAAGTGGCCATGGATTCGCAAGGGCGAAAG; *arx-2* R2, GGGGACAACTTTGTATAATAAAGTTGGAATCAGTTAATAAATGAGTTGGA; *arx-2* R3, GGGGACCACTTTGTACAAGAAAGCTGGGTGAATCAGTTAATAAATGAGTTGGA; dmoesin F, GGGGACAAGTTTGTACAAAAAAGCAGGCTCCATGGACGAAGTGGAAGACGCCC; dmoesin R, GGGGACCACTTTGTACAAGAAAGCTGGGTACATGTTCTCAAACTGATCGAC; dmoesin F1, GGGGACAAGTTTGTACAAAAAAGCAGGCTTCATGGTCTCAAAGGGTGAAG; dmoesin R2, GGGGACCACTTTGTACAAGAAAGCTGGGTAGGATCTTTACATGTTCTCAAAC.

### Statistical tests

Two-tailed Student's *t*-tests were computed using Excel 2013 (Microsoft) without the assumption of equal variance.

### Imaging

Confocal images were taken with a Nikon 2000 U inverted microscope, fitted with a PerkinElmer UltraVIEW ERS 6FE-US spinning disk laser apparatus. Confocal images were processed with ImageJ version 1.48 (NIH). All other worm images were taken by a motorized Zeiss Axioskop equipped with epifluorescence and AxioVision software version 4.8.

Muscle mitochondria were visualized using the *myo-3p::mitoGFP* transgene, Rhodamine 6G dye, or MitoTracker CMXRos dye (Han et al., 2012, 2013). An advantage of the transgene is that muscle mitochondria are specifically labeled, but a disadvantage is that mitoGFP overexpression can cause abnormal mitochondrial morphology and location. The dyes do not appreciably affect mitochondrial morphology or location, although they stain mitochondria in most cells.

### RNA-mediated interference (RNAi)

RNAi was performed by the feeding method (Timmons and Fire, 1998). HT115(DE3) bacterial feeding strains were obtained from the genome-wide library (Kamath et al., 2003). PCR and sequencing (UAB Heflin Center for Genomics Sciences) were used to confirm that strains contained the correct clones. RNAi phenotypes were compared with those of null mutants to determine effectiveness.

### Transgenics

To generate transgenic *C. elegans*, plasmids (5-60 ng/μl) were injected into young adult hermaphrodite gonads. The *myo-3p::mitoGFP* transgene was used to evaluate body wall muscle mitochondria (Labrousse et al., 1999). Multiple independent transgenic lines were analyzed. An extrachromosomal array expressing pan-neuronal mitoGFP (*unc-119p::mitoGFP*) and the synaptic marker RAB-3 (*unc-25p::mCherry::rab-3*) was integrated into the genome by gamma irradiation. The *unc-25p::mCherry::rab-3* transgene was a gift from Dr David Miller (Vanderbilt University). The *pie-1p::vpr-1g* transgenic lines were generated using MosSCI single-copy insertion (*ttTi5605* Mos1 allele, near the center of chromosome II) by Knudra Transgenics. Integrated transgenes were crossed into the *vpr-1(tm1411)* background and maintained as transgenic *vpr-1* mutant homozygotes.

### CRISPR/Cas9

CRISPR/Cas9 methods were performed as previously published (Friedland et al., 2013). DNA template, sgRNA, Cas9, and *myo-3p::mitoGFP* plasmids were injected into *unc-119(ed3)* worms. Progeny were screened for rescue of the *unc-119* movement defect and loss of *myo-3p::mitoGFP*. Individual worms were isolated repeatedly to ensure 100% segregation. PCR and sequencing (UAB Heflin Center for Genomics Sciences) were used to confirm tdTomato insertion. The *clr-1::tdTomato* Cas9 line did not exhibit the fluid accumulation phenotype caused by reduced *clr-1* function (Kokel et al., 1998), indicating that tdTomato fusion does not compromise CLR-1 activity.
